# Association between the *APOE* gene polymorphism and lipid profile and the risk of atrial fibrillation

**DOI:** 10.1186/s12944-021-01551-4

**Published:** 2021-09-29

**Authors:** Xunwei Deng, Jingyuan Hou, Qiaoting Deng, Zhixiong Zhong

**Affiliations:** 1 Department of Research Experimental Center, Meizhou People’s Hospital (Huangtang Hospital), Meizhou, P. R. China; 2 Guangdong Provincial Key Laboratory of Precision Medicine and Clinical Translational Research of Hakka Population, Meizhou, P. R. China; 3 Guangdong Provincial Engineering and Technology Research Center for Molecular Diagnostics of Cardiovascular Diseases, Meizhou, P. R. China; 4 Meizhou Academy of Medical Sciences Cardiovascular Disease Research Institute, Meizhou, P. R. China; 5 Center for Cardiovascular Diseases, Meizhou People’s Hospital (Huangtang Hospital), Meizhou, P. R. China; 6 Meizhou People’s Hospital (Huangtang Hospital), No 63 Huangtang Road, Meijiang District, 514031 Meizhou, China

**Keywords:** Apolipoprotein E, gene polymorphism, atrial fibrillation, Hakka, Southern China

## Abstract

**Background:**

The relationship between the *APOE* gene polymorphism and lipid profiles and atrial fibrillation (AF) remains controversial. The current study purposed to investigate how the *APOE* gene SNPs (rs429358 and rs7412) and lipid profile are associated with the risk for AF among the Hakka population in southern China.

**Methods:**

Finally, 1367 patients were enrolled in this study, including 706 participants with AF (41 ~ 98 years old, 58.64 % male) and 661 non-AF subjects (28 ~ 95 years old, 59.46 % male). The collected data included baseline characteristics, medical history, laboratory tests and echocardiography parameters. A general linear model (two-way analysis of variance (ANOVA)) and Tukey post-hoc tests were applied to identify an *APOE* allele, AF group, and interaction effect on lipid profiles. Logistic regression analysis was performed to identify risk factors for AF.

**Results:**

For AF group, the most common genotype was E3/E3 (53.82 %), followed by E3/E4 (28.19 %), E2/E3 (13.60 %), E4/E4 (1.98 %), E2/E4 (1.84 %) and E2/E2 (0.57 %). The two-way ANOVA followed by the Tukey procedure showed the following: the lipid levels depended significantly on AF and *APOE* allele groups for TG, TC, LDL-C and Apo-B (all *P* < 0.001), and statistically significant interactions between AF and *APOE* allele were observed in the above 4 variables (all *P* < 0.05). Multivariate regression analysis indicated that age ≥ 65years (*P* < 0.001), high diastolic blood pressure (DBP ≥ 90mm Hg, *P* = 0.018), a high levels of total cholesterol (TC ≥ 5.2mmol/L, *P* < 0.001) and triglyceride (TG ≥ 1.7mmol/L, *P* = 0.028), but not the two SNPs of the *APOE* gene (rs7412 and rs429358) (OR 1.079, *P* = 0.683), were significant independent risk factors for AF in the study population.

**Conclusions:**

The principal findings of this study showed that individuals at high risk for AF were those over 65 years of age, higher DBP as well as high levels of TC and TG among the southern China Hakka population. The levels of TG, TC, LDL-C and Apo-B depended significantly on AF and *APOE* allele groups, and statistically significant interactions between AF and *APOE* allele were observed in the above 4 variables, although the *APOE* gene SNPs (rs429358 and rs7412) were no significant risk for AF incidence. Further investigation is needed to elucidate whether other SNPs of the *APOE* gene have a bearing on AF incidents.

## Background

Atrial fibrillation (AF) represents the most prevalent type of cardiac arrhythmia. The prevalence of AF has rapidly increased and is estimated to surpass 25 million cases by 2045 in China [1]. Since AF significantly contributes to mortality and health burden worldwide, it is of vital significance to study its underlying pathophysiological mechanisms. Over the past few decades, plenty of research works have investigated the clinical features and mechanisms of AF [2, 3]. The reported results indicated that the AF mechanism might be related to oxidative stress [4], inflammation [5] as well as electrical and structural remodeling of the atria [6]. Based on the close association between AF and other cardio-cerebrovascular diseases (e.g., heart failure, thromboembolism and stroke), there might be a potential correlation between their pathomechanisms. Hence, exploring the potential link between AF and the levels of blood lipid, which represent a well-established risk factor for cardio-cerebrovascular events, may provide a new perspective to identify the mechanism of AF and optimize the management of AF patients.

Dyslipidemia is deeply at risk for cardiovascular diseases [7, 8]. The alteration of lipid metabolism is a representative feature in the incident and evolvement of atherosclerosis [9]. Long-term hyperlipidemia damages the artery intima and promotes atherosclerosis through the inflammatory-fibroproliferative reaction, eventually leading to severe cardiovascular events, such as coronary heart disease, heart failure and stroke. There is increasing evidence that the occurrence of AF is linked to the lipid profile, including the epicardial fat [10], pericardial fat, intrathoracic fat, abdominal visceral fat and body mass [11]. In the epicardial region of the heart, epicardial progenitor cells play a key role in the occurrence of arrhythmic events, which can be transformed into adipocytes under various stimuli [12].

Apolipoproteins play a leading role in lipid transport and in maintaining the balance of lipid metabolism [13]; they are closely related to lipid disorders. Apolipoprotein E (ApoE) is a polymorphic protein and generates six genotypes (E3/E3, E3/E4, E2/E3, E4/E4, E2/E4 and E2/E2). These genotypes are determined by rs429358 and rs7412 SNPs of the *APOE* gene, which exists in three major isoforms and has 3 allelic variants (ε2, ε3 and ε4) [13]. In southern China, approximately 65 % of the Hakka population carry a common E3/E3 genotype [14]. Previous studies have revealed that the *APOE* ε4 allele is an established genetic risk factor for coronary heart disease (CHD) [15], atherosclerosis and Alzheimer’s disease [16] and influences the development of CHD. In addition, CHD patients with the *APOE* ε4 carrier were significantly correlated with the incidence of ischemic stroke [17]. In patients with Alzheimer’s disease, *APOE* ε4 carrier was discovered to lead to a significantly faster disease progression [18]. Moreover, evidence suggests that the long-term use of antipsychotics in *APOE* ε4 carriers can also severely decrease cognitive function [19]. However, the association between AF and the *APOE* genotype has not been adequately investigated.

The present study aimed to investigate the possible potential role of the *APOE* genotype distribution and lipid profile in AF in the Hakka population of southern China. The *APOE* gene polymorphism may impact AF development by altering the lipid profile. These findings may provide a practical way to enhance the primary health care strategy of AF patients.

## Subjects and methods

### Ascertainment of AF

Two experienced cardiologists analyzed the standard twelve-lead resting electrocardiography (ECG) to ascertain the AF incidence. The ECG was analyzed back-to-back according to the European Society of Cardiology / European Association for Cardio-Thoracic Surgery (ESC/EACTS) guidelines: (1) absence of distinct P waves; (2) irregular R-R intervals; (3) an irregular ventricular response; (4) atrial cycle length, which is the time interval between two atria activation, of < 200 ms.

Based on the 2020 ESC/EACTS guidelines for AF management [20], AF is classified to: paroxysmal, persistent, longstanding persistent and permanent, depending on the frequency and duration. Paroxysmal AF was defined as AF with spontaneous termination or that requires intervention to restore the sinus rhythm within 7 days, while persistent AF lasts longer than 7 days.

### Subjects

**Fig. 1:**
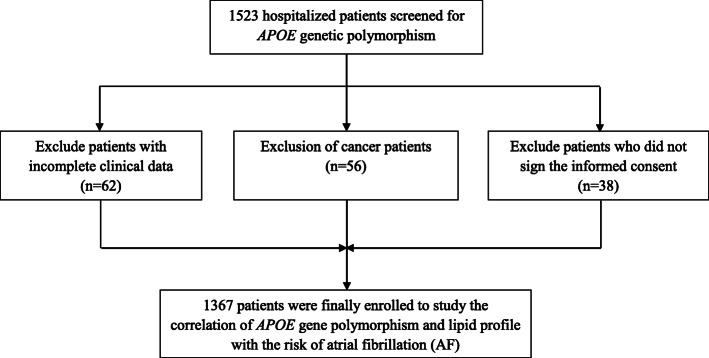
Flow chart of investigating the correlation of *APOE* gene polymorphism and lipid profile with the risk of AF

A total of 1523 participants who visited Meizhou People’s Hospital (Huangtang Hospital), Guangdong, China, from May 2016 to April 2021 were included in the study. The main inclusion criteria for the AF group were: (1) confirmed diagnosis of AF by attending physicians; (2) age 18 years or above; (3) complete information of the clinical laboratory data. The exclusion criteria were: (1) history of malignant tumors or autoimmune diseases; (2) severe infection, tuberculosis or metabolic syndrome; (3) major diseases that affect the metabolism of inflammatory factors and blood lipids; (4) severe hepatic or renal diseases; (5) missing information of the baseline variables or ECG; (6) currently receiving lipid-lowering treatment or agents except statins; (7) unable to fully participate in the survey for other reasons. Subjects with no evidence of AF on history or electrocardiogram were included in the non-AF group. Of these, participants with incomplete clinical data (*n* = 62), those who did not sign the informed consent (*n* = 38) and those with a history of malignant tumors (*n* = 56) were excluded from the study. Finally, a total of 1367 participants met the inclusion criteria (showed in Fig. 1).

All the participants had complete medical records, including alcohol intake, smoking status, *APOE* genotyping, systolic pressure, diastolic pressure and lipid profile outcomes. Hypertension was defined as a blood pressure (BP) exceeding 140/90 mm Hg as the mean of 3 independent measures or a current antihypertensive therapy [21]. Diabetes was diagnosed as a 2 h post-load glucose value of ≥ 200 mg/dl (11.1 mmol/l), a fasting blood glucose ≥ 126 mg/dl (7 mmol/L) or a current treatment with antidiabetic medications. According to the Chinese adult dyslipidemia prevention guide (2016 edition), high TC was defined as total cholesterol (TC) ≥ 5.2 mmol/L, high TG was defined as triglycerides (TG) value ≥ 1.7 mmol/L, high LDL-C was defined as serum low-density lipoprotein-cholesterol (LDL-C) concentrations greater than 3.4 mmol/L, and low HDL-C was defined as a value of high-density lipoprotein-cholesterol (HDL-C) < 1.0 mmol/L [22]. The normal levels of the following indexes exhibited the following range: Apolipoprotein A1 (Apo A1) from 1 to 1.6 g/L; Lipoprotein a (Lp-a) from 0 to 30 mg/mL; and Apolipoprotein B (Apo B) from 0.6 to 1.1 g/L [23]. Atherosclerosis was defined as a complex pathological change that involves an excessive inflammatory response, hyperlipidemia, thrombus formation following injurious stimuli, plaque evolution and destabilization, leading to myocardial ischemia as well as necrotic or coronary artery disease. Heart failure was defined as a clinical syndrome with typical symptoms (e.g., breathlessness, fatigue), which may be accompanied by other manifestations (e.g., hypoxemia, pneumonedema) due to a structural and/or functional cardiac abnormality.

The study protocol was performed according to the ethical guidelines of the 1975 Declaration of Helsinki and approved by the institutional review board and ethical committee of the Meizhou Peoples’ Hospital (No. MPH-HEC 2021-C-19). All patients provided written informed consent before taking part in the study.

### Sample collection

All fasting blood sample was divided into two aliquots: one in an ethylenediaminetetraacetic acid (EDTA) vial and the other in a plain vial. DNA was extracted from the sample with EDTA, while the serum was used for biochemical analysis. The samples were stored at -20 °C until being processed.

### Lipid analysis and clinical data acquisition

Lipid analysis were performed for serum samples in two groups. The Beckman Coulter clinical chemistry autoanalyzer AU5400 was used to assay the TC, TG, HDL-C, LDL-C, apolipoprotein A1 (Apo-A1) and apolipoprotein B (Apo-B). Besides, the parameters of the alcohol intake and smoking status of participants were categorized into never, former or current. The systolic and diastolic pressure values were measured twice in a sitting position using a sphygmomanometer at 5-minute intervals after resting for at least 5 min. In addition, the following data was collected: (1) demographic data (age, sex); (2) clinical laboratory data on the liver and kidney function, including alanine aminotransferase (ALT), aspartate aminotransferase (AST), urea nitrogen (UN), serum creatinine (Scr), uric acid (UA), C-reactive protein (CRP) and white blood cell (WBC); (3) echocardiographic results, including the left atrium diameter (LAD), left ventricular end-diastolic diameter (LVDd), left ventricular end-systolic diameter (LVSd) and left ventricular ejection fractions (LVEF); (4) personal medical history during a medical appointment. We gathered all demographic and clinical results from our hospital’s computerized medical recording system.

### ***APOE*** genotyping

Genomic DNA was isolated from 2 ml of whole peripheral blood samples of both groups using the QIAamp DNA Blood Mini Kit (Qiagen, Germany) following the manufacturer’s protocol. A Nanodrop 2000 TM Spectrophotometer was used for DNA concentration and purity, with an A260/280 ratio > 1.7 considered to be qualified. The two SNPs of rs429358 (E4) and rs7412 (E2) of the *APOE* gene were determined using a commercial kit (LOT no. B50201013A, Sinochips Bioscience Co., Ltd, Zhuhai, Guangdong, China). Protocol for polymerase chain reaction (PCR) was as follow: 50 °C for 2 min, pre-denaturation at 95 °C for 15 min, followed by 45 cycles comprising of denaturing at 94 °C for 30 s and annealing at 65 °C for 45 s. The amplified products were subsequently dispensed into a hybridization reaction chamber. The genotype was revealed using an *APOE* gene chip assay (Sinochips Bioscience Co., Ltd, Zhuhai, China) according to the manufacturer’s instructions. For the genotyping quality control, blank tubes without DNA were included in all the SNPs that were analyzed as the negative control and confirmed by duplicate analysis of 10 % of samples. Figure 2 shows the microarray results of the kit for six types of the *APOE* genotype.

**Figure:**
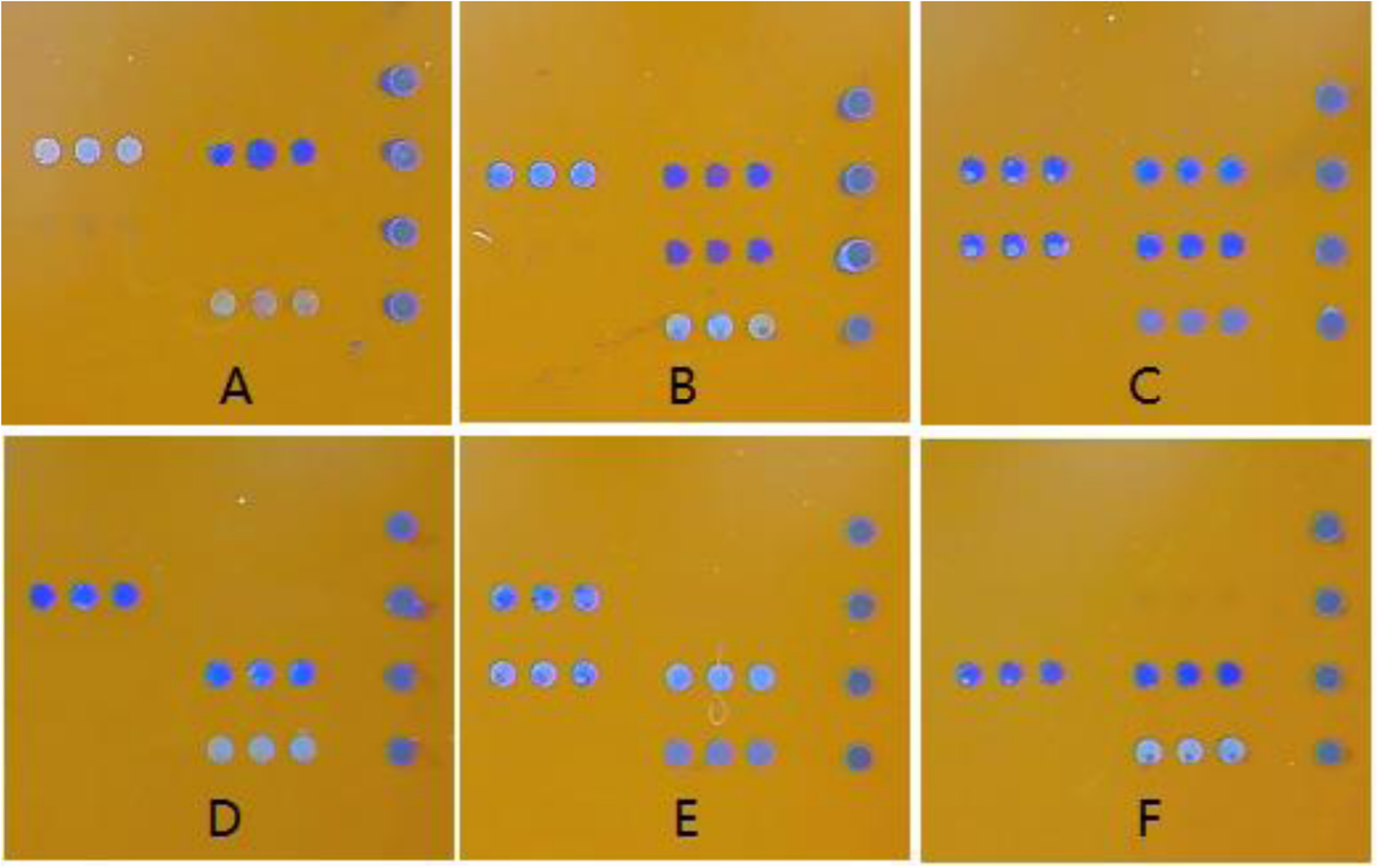
Microarray results of *APOE* genotypes. A: E2/E2, B: E2/E3, C: E2/24, D: E3/E3, E: E3/34, F: E4/E4

Linkage disequilibrium (LD) figure and values were calculated with Haploview 4.2 (http://www.broadinstitute.org/scientific-community/science/programs/medical-andpopulation- genetics/haploview/haploview).

### Statistical analysis

Statistical analysis was assessed with the IBM SPSS v.19 software. The Hardy-Weinberg equilibrium of the allele and genotype between-group differences was performed with the Chi-square test. The Kaplan–Meier method was used to evaluate the data normality. Measurement variables were represented as mean values ± standard deviation (SD) and compared via unpaired Student’s t-test or Mann-Whitney U test as appropriate. Categorical variables were expressed as a number (percent) and analyzed using the Chi-square test. A two-way ANOVA followed by Tukey post-hoc test, which included the factors *APOE* allele and AF, was conducted to assess the effect of those on the levels of lipid profiles. Logistic regression analysis was conducted to investigate the associations of the risk factors related to the occurrence of AF and potential covariates. Two-sided *P*-value below 0.05 was regarded to be significant.

## Results

### Clinical characteristics of the participants

A total of 1367 individuals were included, among which 706 participants (292 women and 414 men) were patients with paroxysmal or persistent AF, aged 41 ~ 98 years old and 661 participants (268 women and 393 men) without AF as controls, with an average age of (67.11 ± 11.92). Table 1 lists the baseline characteristics and medical history of all the participants. There was a significant difference in age, diastolic blood pressure (DBP), CAD and HF incidence between the AF and control groups. Patients with AF were older than those in the control group, had higher DBP, and had a higher incidence of CAD and HF (all *P* < 0.001). Regarding the medical history, the prevalence of diabetes and hypertension was 29.18 % and 60.06 %, respectively, in the AF group and 28.44 % and 57.34 %, respectively, in the control group. However, none of the prevalence in diabetes and hypertension differed across the two groups. Furthermore, no significant difference was found across the two groups regarding the factors of sex, systolic blood pressure (SBP), *APOE* ε4 carrier, smoking status and alcohol intake. In the received medication, patients with AF took more ACEIs, BBs, MRAs, diuretics, and digoxin (all *P* < 0.001).

**Table 1:** Baseline characteristics, medical history and drug therapy of the study participants

Variables		AF patients (*n* = 706)	Controls (*n* = 661)	*P*
Baseline characteristics, n (%)				
Age ≥ 65y		575(81.44)	396(59.91)	**< 0.001**
Male		414(58.64)	393(59.46)	0.783
SBP ≥ 140mm Hg		329(46.60)	307(46.44)	0.954
DBP ≥ 90mm Hg		242(34.28)	173(26.17)	**< 0.001**
*APOE*-ε4 carrier		226(32.01)	180(27.23)	0.053
Alcohol intake				
Never		677(95.89)	630(95.31)	0.175
Former		15(2.12)	9(1.36)	
Current		14(1.98)	22(3.33)	
Smoking status				
Never		542(76.77)	498(75.34)	0.101
Former		89(12.61)	70(10.59)	
Current		75(10.62)	93(14.07)	
Medical history, n (%)				
Hypertension		424(60.06)	379(57.34)	0.307
Diabetes		206(29.18)	188(28.44)	0.764
CAD		390(55.24)	223(33.74)	**< 0.001**
HF		294(41.64)	63(9.53)	**< 0.001**
Drug Therapy, n (%)				
Statin		16(2.27)	12(1.82)	0.556
ACEIs		190(26.91)	111(16.79)	**< 0.001**
ARBs		148(20.96)	149(22.54)	0.479
BBs		302(42.78)	179(27.08)	**< 0.001**
MRAs		275(38.95)	53(8.02)	**< 0.001**
Digoxin		197(27.90)	24(3.63)	**< 0.001**
Diuretics		324(45.89)	91(13.77)	**< 0.001**

Values for age expressed as mean ± SD

SBP: systolic blood pressure

DBP: diastolic blood pressure

CAD: coronary artery disease

HF: heart failure

ACEIs: angiotensin-converting enzyme inhibitors

ARBs: angiotensin receptor blockers

BBs: β-receptor blockers

MRAs: mineralocorticoid antagonists

The echocardiography results and laboratory data of the two groups are shown in Table 2. Overall, the patients in the AF group had significantly larger LAD, LVDd and LVSd, along with a significantly lower LVEF (all *P* < 0.001). Regarding the levels of lipid profiles, the subjects in the AF group also had significantly higher levels of all parameters (all *P* < 0.001), except for HDL-C and Apo-A1, compared with the control group. Nevertheless, the controls had significantly lower levels of ALT, AST, UN, Scr and UA (all *P* < 0.001). Moreover, no significant differences in the CRP and WBC across the two groups.

**Table 2:** Echocardiography results and laboratory data of the study participants

Variables	AF patients (*n* = 706)	Controls (*n* = 661)	*P*
Echocardiography results			
LAD, mm	40.23 ± 8.40	31.34 ± 4.63	**< 0.001**
LVDd, mm	46.82 ± 8.44	43.95 ± 5.75	**< 0.001**
LVSd, mm	33.85 ± 9.17	29.44 ± 6.03	**< 0.001**
LVEF, %	54.13 ± 12.84	62.20 ± 8.37	**< 0.001**
Laboratory data			
TC ≥ 5.2mmol/L, n (%)	178(18.13)	48(7.26)	**< 0.001**
TG ≥ 1.7mmol/L, n (%)	167(23.65)	96(14.52)	**< 0.001**
HDL-C < 1mmol/L, n (%)	191(27.05)	166(25.11)	0.414
LDL-C ≥ 3.4mmol/L, n (%)	109(15.44)	18(2.72)	**< 0.001**
Apo-A1 ≥ 1.6 g/L, n (%)	38(5.38)	48(7.26)	0.153
Apo-B ≥ 1.1 g/L, n (%)	99(14.02)	26(3.93)	**< 0.001**
ALT, U/L	32.42 ± 25.54	26.80 ± 20.05	**< 0.001**
AST, U/L	42.49 ± 43.66	29.83 ± 27.88	**< 0.001**
UN, mmol/L	8.24 ± 5.06	6.46 ± 3.30	**< 0.001**
Scr, umol/L	125.70 ± 85.70	102.51 ± 70.39	**< 0.001**
UA, umol/L	412.45 ± 145.89	325.74 ± 110.82	**< 0.001**
CRP, mg/L	46.59 ± 57.46	41.81 ± 46.65	0.302
WBC, 10^9^/L	9.53 ± 4.55	9.04 ± 4.44	0.563

LAD: left atrium diameter

LVDd: left ventricular end-diastolic diameter

LVSd: left ventricular end-systolic diameter

LVEF: left ventricular ejection fractions

TC: total cholesterol

TG: triglyceride

HDL-C: high-density lipoprotein cholesterol

LDL-C: low-density lipoprotein cholesterol

Apo-A1: apolipoprotein A1

Apo-B: apolipoprotein B

ALT: alanine aminotransferase

AST: aspartate aminotransferase

UN: urea nitrogen

Scr: serum creatinine

UA: uric acid

CRP: C-reactive protein

WBC: white blood cell

### ***APOE*** genotype and allele frequencies

The distribution of the *APOE* genotype and allele in the AF and control group is listed in Table 3. In the AF group, the predominant genotype was E3/E3 (53.82 %), followed by E3/E4 (28.19 %), E2/E3 (13.60 %), E4/E4 (1.98 %), E2/E4 (1.84 %) and E2/E2 (0.57 %). In the control group, the frequencies of genotypes E3/E3, E3/E4, E2/E3, E4/E4, E2/E4 and E2/E2 were 57.19, 22.84, 14.83, 2.27, 2.12 and 0.76 %, respectively.

**Table.3:** The distributions of *APOE* genotypes and alleles in patients with AF and control groups

	AF patients (*n* = 706)	Controls (*n* = 661)	χ^2^	*P*
Genotype	*n* = 706	*n* = 661		
E2/E2	4(0.57)	5(0.76)	5.32	0.379
E2/E3	96(13.60)	98(14.83)		
E2/E4	13(1.84)	14(2.12)		
E3/E3	380(53.82)	378(57.19)		
E3/E4	199(28.19)	151(22.84)		
E4/E4	14(1.98)	15(2.27)		
Allele	*n* = 1412	*n* = 1322		
ε2	117(8.29)	122(9.23)	3.01	0.222
ε3	1055(74.72)	1005(76.02)		
ε4	240(16.99)	195(14.75)		
Hardy-Weinberg equilibrium	χ^2^ *=* 8.05, *P =* 0.090	χ^2^ *=* 1.38, *P =* 0.849		

The *APOE* genotypes were divided into the following three subgroups according to the alleles: ε2 (E2/E2 and E2/E3), ε3 (E3/E3) and ε4 (E2/E4, E3/E3, and E3/E4). The frequencies of the *APOE* alleles ε2, ε3 and ε4 were 8.29 %, 74.72 % and 16.99 %, in AF patients and 9.23 %, 76.02 % and 14.75 % in the control subjects, respectively. In both groups, the distribution of the *APOE* polymorphism genotypic frequencies followed the Hardy-Weinberg equilibrium. There was no evidence about the frequency of the *APOE* genotypes and alleles differed across the two groups. (*P* = 0.379 and *P* = 0.222, respectively).

### Linkage disequilibrium

**Fig. 3:**
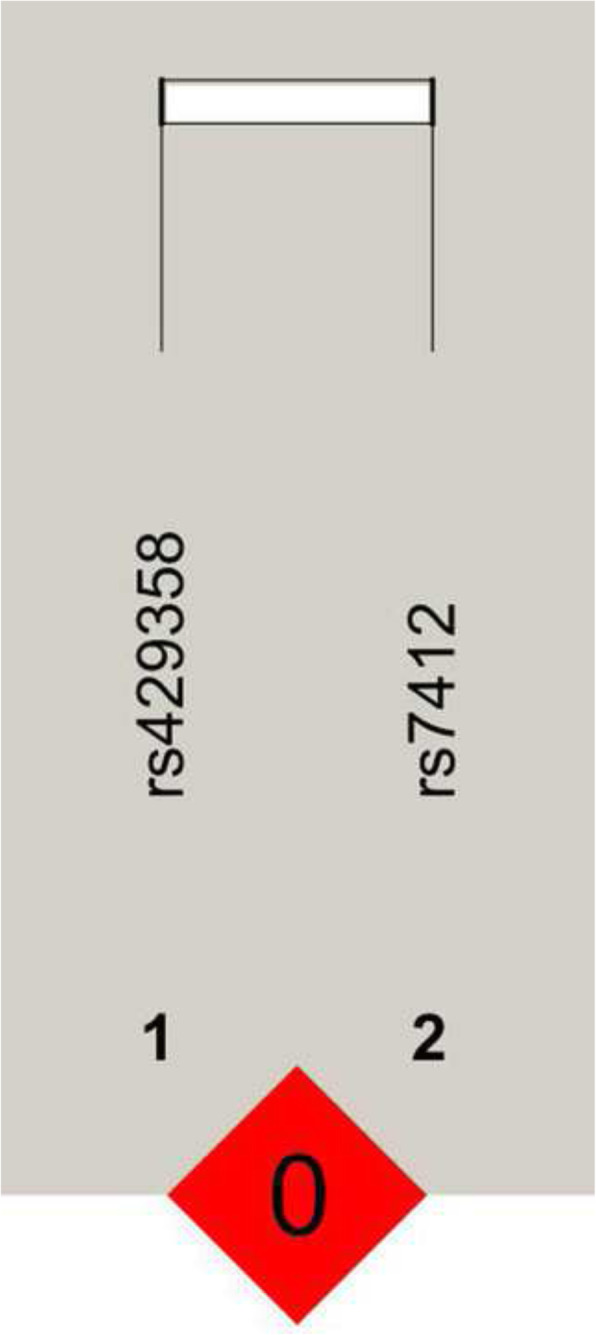
Linkage disequilibrium (LD) values of the SNPs

The LD between the *APOE* rs429358 and rs7412 polymorphisms was evaluated (Fig. 3), the two SNPs were not in perfect LD (r^2^ = 0.018). So the analysis of all polymorphisms was investigated.

### ***APOE*** allele, AF and lipid levels

**Fig. 4:**
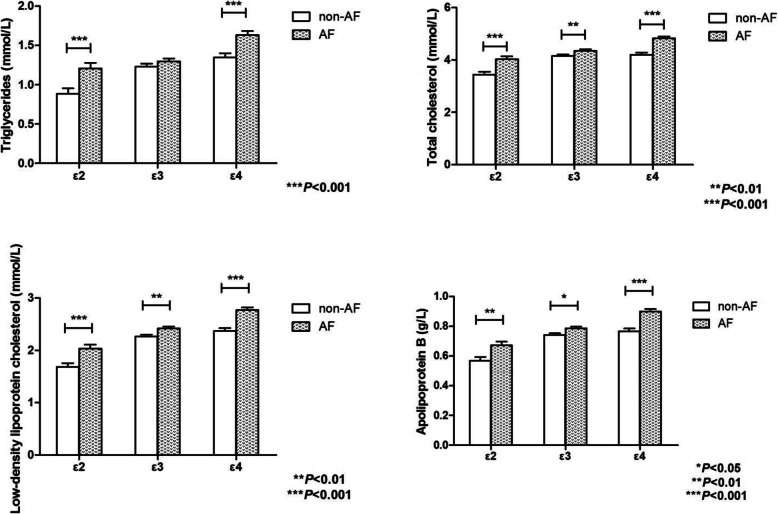
The effect of *APOE* allele groups (ε2, ε3 and ε4) and AF groups (AF vs. non-AF) on the levels of TG, TC, LDL-C and Apo-B

The differences in lipid profile levels as related to the *APOE* allele groups (ε2, ε3 and ε4) and AF were shown in Table 4. Patients carrying the E2/E4 genotype (*n* = 27) were excluded for the reverse influence in lipid metabolism by ε2 and ε4 alleles. The lipid levels depended significantly on AF and *APOE* allele groups for TG, TC, LDL-C and Apo-B (all *P* < 0.001), and statistically significant interactions between AF and *APOE* allele were observed in the above 4 variables (TG, *F* = 4.478, *P* = 0.012; TC, *F* = 6.189, *P* = 0.002; LDL-C, *F* = 3.717, *P* = 0.025; Apo-B, *F* = 4.529, *P* = 0.011, respectively). The result also showed that there was a significant difference in levels of HDL-C between *APOE* allele groups (*P* = 0.018) but insignificant in AF (*P* = 0.264). On finding this, a Tukey procedure was performed to test the simple main effects. The results revealed that TG levels for ε2 carrier with AF were higher than that for ε2 carrier without AF (*F* = 10.698, *P* < 0.001) (Fig. 4). Similar results were obtained concerning TG levels for ε4 carrier (*F* = 15.118, *P* < 0.001). Additionally, a Tukey post-hoc test of the simple main effects showed that regarding TC, LDL-C and Apo-B levels, patients with AF were significantly higher than those without AF in all 3 *APOE* allele groups (all *P* < 0.05).

**Table 4:** Lipid levels and the results of two-way ANOVA with AF groups and the three *APOE* alleles

	*APOE* allele groups			AF groups		ANOVA interaction (AF and *APOE* alleles)	
	ε2(*n* = 203)	ε3(*n* = 758)	ε4(*n* = 379)	AF (*n* = 693)	Non-AF (*n* = 647)	*F*	*P*
TC (mmol/L)	3.737(0.075;3.590–3.884)	4.253(0.039;4.177–4.329)	4.512(0.055;4.404–4.621)	4.404(0.047;4.312–4.496)	3.930(0.048;3.386–4.025)	6.189	**0.002**
TG (mmol/L)	1.045(0.050;0.947–1.143)	1.264(0.026;1.214–1.315)	1.490(0.037;1.417–1.562)	1.380(0.031;1.319–1.441)	1.153(0.032;1.090–1.216)	4.478	**0.012**
HDL-C (mmol/L)	1.275(0.027;1.223–1.327)	1.240(0.014;1.213–1.267)	1.188(0.020;1.149–1.226)	1.248(0.017;1.215–1.280)	1.221(0.017;1.187–1.254)	0.214	0.808
LDL-C (mmol/L)	1.857(0.053;1.753–1.962)	2.340(0.028;2.286–2.394)	2.566(0.039;2.488–2.643)	2.406(0.033;2.340–2.471)	2.103(0.034;2.035–2.170)	3.717	**0.025**
Apo-A1 (g/L)	1.066(0.023;1.020–1.111)	1.074(0.012;1.050–1.097)	1.066(0.017;1.033–1.099)	1.049(0.014;1.021–1.077)	1.088(0.015;1.059–1.117)	0.264	0.768
Apo-B (g/L)	0.620(0.017;0.587–0.654)	0.763(0.009;0.745–0.780)	0.833(0.013;0.808–0.857)	0.785(0.011;0.764–0.806)	0.692(0.011;0.670–0.713)	4.529	**0.011**

Mean (SD; range)

AF: atrial fibrillation

TC: total cholesterol

TG: triglyceride

HDL-C: high-density lipoprotein cholesterol

LDL-C: low-density lipoprotein cholesterol

Apo-A1: apolipoprotein A1

Apo-B: apolipoprotein B

### Assessing risk factors for AF with logistic regression

A multiple logistic regression test was used to evaluate the independent effect of the categorical variables (age, DBP, *APOE*-ε4 carrier, TC, TG, LDL-C, Apo-B) on the risk of AF (Table 5). These clear associations between categorical variables and AF were seen on logistic regression analysis with the non-adjusted model. After adjusting for the quantitative variables, such as the echocardiography results (LAD, LVDd, LVSd, and LVEF), laboratory data (ALT, AST, UN, Scr and UA), and categorical variables including concomitant drugs (ACEIs, BBs, MRAs, digoxin and diuretics), CAD and HF, the consequences indicated that age ≥ 65 years (OR 2.848, 95 % CI 1.894–4.283, *P* < 0.001), high DBP (OR 1.556, 95 % CI 1.080–2.240, *P* = 0.018), high TG (OR 1.596, 95 % CI 1.053–2.419, *P* = 0.028) and high TC (OR 3.879, 95 % CI 2.003–7.513, *P* < 0.001) represented independent risk factors for AF, but not the *APOE* gene SNPs (rs7412 and rs429358) (OR 1.079, 95 % CI 0.748–1.558, *P* = 0.683).

**Table 5:** Logistic regression analysis for risk factors of AF in the study participants

Parameters	No. subjects	*P*	Non-adjusted OR(95 % CI)	*P*	Adjusted OR(95 % CI)
Age					
(< 65y)	396		(Ref.)		(Ref.)
(≥ 65y)	971	**< 0.001**	2.937(2.298–3.754)	**< 0.001**	2.848(1.894–4.283)
DBP					
(< 90mm Hg)	952		(Ref.)		(Ref.)
(≥ 90mm Hg)	415	**< 0.001**	1.471(1.166–1.857)	**0.018**	1.556(1.080–2.240)
*APOE*-ε4 carrier					
no	961		(Ref.)		(Ref.)
yes	406	0.053	1.258(0.997–1.588)	0.683	1.079(0.748–1.558)
TC					
(< 5.2mmol/L)	1141		(Ref.)		(Ref.)
(≥ 5.2mmol/L)	226	**< 0.001**	4.305(3.066–6.045)	**< 0.001**	3.879(2.003–7.513)
TG					
(< 1.7mmol/L)	1104		(Ref.)		(Ref.)
(≥ 1.7mmol/L)	263	**< 0.001**	1.823(1.382–2.406)	**0.028**	1.596(1.053–2.419)
LDL-C					
(< 3.4mmol/L)	1240		(Ref.)		(Ref.)
(≥ 3.4mmol/L)	127	**< 0.001**	6.522(3.913–10.872)	0.596	1.308(0.485–3.527)
Apo-B					
(< 1.1 g/L)	1242		(Ref.)		(Ref.)
(≥ 1.1 g/L)	125	**< 0.001**	3.983(2.550–6.222)	0.459	1.380(0.589–3.233)

OR in adjusted model was adjusted for LAD, LVDd, LVSd, LVEF, ALT, AST, UN, Scr, UA, CAD, HF, use of ACEIs, BBs, MRAs, digoxin and diuretics

OR: odds ratio; CI: confidence interval. Other abbreviations see in Tables 1 and 2

## Discussion

AF represents the most common cardiac arrhythmia caused by atrial electrical remodeling, which leads to the inability to coordinate the contraction and relaxation of the atrium and constitutes a major cause of morbidity. It has been found to tightly link with a higher risk of hospitalization, cardiovascular mortality and cognitive impairment in individuals [24]. AF is also a major health care challenge with an increasing burden with age [25]. Evidence from previous studies suggests that many genes and/or environmental factors may have a combined influence on the occurrence and evolvement of AF [26].

Hypertension and AF frequently coexist, not only because hypertension increases the incidence of new-onset of AF, but also because those two entities share common risk factors and conditions that increase the incidence of both. Hypertension increases the risk of AF occurrences through hemodynamic and non-hemodynamic mechanisms. Uncontrolled high blood pressure increases left ventricular wall load by increasing left ventricular wall tension [27]. Over time, cardiac hypertrophy becomes morbid, and the heart can no longer meet the increasing mechanical workload, resulting in dilation [28]. Diastolic dysfunction causes myocardial fibrosis [29], which may lead to left atrial structure and function are altered [30]. Specifically, structural remodeling leads to electrolytic dissociation and local conduction heterogeneity between muscle bundles, which promotes the occurrence and perpetuation of AF [31].

Many research studies were conducted to discover promising genetic susceptibility markers of AF. With the progress in genome-wide association (GWAS) analysis in recent years, genomics has made considerable progress in the prediction of complex human diseases, such as CAD and AF [32]. To date, over 50 pathogenic gene mutations have been revealed to cause AF, which mainly involve cellular signal molecules [33], myocardial structural proteins [34], ion [35] and gap junction channels [36]. Hsu et al. [34] reported that subjects with AF carrying the ALDH2*2 polymorphism showed more severe oxidative stress in their atria compared with non-carriers and suggested that *ALDH2* may protect from AF-related remodeling. In a single-center retrospective study, Okamura and colleagues [36] analyzed the SNPs reported in a GWAS analysis of patients with persistent AF and demonstrated that the *GJA1* SNP rs1015451 has increased the risk for a higher heart rate during AF. Furthermore, Liu et al. [37] reported that the *ACE2* SNP rs4646188 was related to a larger left atrial end-systolic diameter and associated with a higher AF risk among Uygur patients. A recent retrospective study enrolled 155 Mongolian descent patients and revealed that the *ABCB1* gene polymorphism may help to achieve a rational drug utilization due to the rs1128503 locus variations, which were linked to rivaroxaban concentration [38]. A Mendelian randomization study by Wang et al. [39] revealed the circulating GDF-15 levels to be significantly related to the increased risk of CAD and AF, which may optimize strategy for AF treatment.

As a well-documented risk factor for cardiovascular disease, obesity accounts for nearly one-fifth of patients with AF [40]. It has been highlighted by demographic surveys to be an important component of the AF prevention and management strategies [41]. A previously published study revealed that weight reduction through bariatric surgery may reduce the risk for AF development by approximately one-third among obese subjects [42]. Since the *APOE* gene has a prominent role in lipoprotein transformation and metabolism, there may be a potential interrelation that probably exists in the *APOE* gene polymorphism and AF. After followed for 2 years, a prospective cohort study found that the interaction between permanent non-valvular AF and *APOE* ɛ4 genotype was linked with a higher risk of cognitive impairment [43], which suggested that *APOE* ɛ4 genotype may participate in the occurrence of AF through some mechanism in Alzheimer’s disease. Besides, after whole-exome-based transcriptome analysis of the transcript profiles in the left and right atrium, Tsai et al. reported that AF was associated with the upregulation of the *APOE* gene, provided evidence that the *APOE* gene may be associated with AF incidence [44]. In recent years, epicardial fat has been identified to be closely linked to the occurrence, severity and even recurrence of AF through several epidemiological and clinical studies [10]. As a type of visceral adipose tissue, epicardial fat is closely adjacent to the coronary artery and myocardium. In a pathological state, epicardial adipose tissue may lead to atrial electrophysiological alteration through infiltration, which may cause AF. Huang et al. conducted a series of studies to assess ApoE expression levels in different adipose tissue depots from multiple species, indicated that in baboons, evaluation of ApoE expression is higher in epicardial adipocytes [45]. *APOE* ε4 allele may be related to epicardial fat thickening, which is closely linked with AF occurrence. In this study, the analysis results indicated that the lipid levels depended significantly on AF and *APOE* allele groups for TG, TC, LDL-C and Apo-B (all *P* < 0.001), and statistically significant interactions between AF and *APOE* allele were observed in the above 4 variables, which indicates the potential correlations in the lipid profile, *APOE* genotypes and pathogenesis of AF, although the *APOE* gene SNPs (rs429358 and rs7412) were not significant independent risk factors for AF incidence. Based on the complex mechanism of AF, the *APOE* gene polymorphism may only participate in some links cooperatively, rather than being the main influencing factor in the development of AF. Moreover, other SNPs of the *APOE* gene were not included in this study. A large-scale set of participants is required to further investigate the association between the *APOE* gene and AF.

Multiple risk factors have been well-documented for the prevalence of AF. However, the relationship between the lipid profile and development of AF remains uncertain. In a racially diverse, community-based, prospective cohort study, high levels of HDL-C were found to reduce the risk of AF, while high TG concentration was revealed to higher risk for AF, and TC and LDL-C were not linked to the risk of AF [46]. An observational study provided evidence that compared with subjects without AF, patients with AF had lower levels of TC, LDL-C and HDL-C, and suggested that low HDL-C levels were significantly associated with a new occurrence of AF [47]. In contrast, after an average of 3.5 years of follow-up, the authors revealed that the HDL-C and TG levels were not correlated with new-onset AF [48]. Inconsistent with the above-mentioned results, the current study showed that high levels of TC and TG, but not HDL-C, were significant independent risk factors for AF. The inconsistent outcomes of different studies may be caused by regional and ethnic discrepancies in the study subjects, socio-demographic composition, sample size or complex environment-genotype-phenotype interactions. Previous studies have confirmed that long-term hyperlipidemia may influence cardiac function and electrophysiological activity [49, 50], involving the alteration of the membrane lipid bilayer as well as modulation of intracellular calcium ions and isoform expression patterns of the myosin heavy chain [51]. The alternate expression in a series of crucial proteins has confirmed the negative effect of hyperlipidemia on the heart [52, 53]. Main mechanisms include the following. First, high levels of TC lead to systemic oxidative stress and a proinflammatory state [54]. There are a number of evidence that point to a role for inflammatory processes in the pathophysiology of AF. Inflammatory effects on the atrial myocardium may facilitate arrhythmogenesis [55]. Oxidative stress is implicated in the pathophysiology of atrial remodeling, which promotes the development of atrial fibrillation [4]. Second, hyperlipidemia downregulates autophagy and promotes the apoptosis of cardiomyocytes, which may be responsible for the loss of cardioprotection [56]. Third, Ca^2+^/ calmodulin-dependent protein kinase II (CaMKII) acts as a crucial factor in regulating the normal electrophysiology and structure of cardiomyocytes. Under hyperlipidemia conditions, the increased expression of CaMKII in cardiomyocytes induces their electrical remodeling and triggers arrhythmia [57]. All these mechanisms were closely associated with the incidence of AF. Notably, there was no significant association between LDL-C and AF after adjusting for other parameters (OR: 1.308, 95 % CI: 0.485–3.527, *P* = 0.596). The relationship between LDL-C and AF was unexpected and no clear mechanism could be presented. Several possible mechanisms may account for the result. First, clinical and subclinical hyperthyroidism may be potentially responsible for the LDL-C reduction as well as the promotion of AF. Thyroid hormone promotes cholesterol catabolism and excretion, lowers circulating levels of LDL-C. Hyperthyroidism is also known to be an independent risk factor for AF. Unfortunately, the lack of thyroid functions data in the current study made it difficult to confirm the association. Second, inflammation may be an important link between cholesterol and AF. Due to the action of inflammatory cytokines, the levels of TC, LDL-C and HDL-C were decreased, while TG was increased in inflammation [58]. Therefore, low cholesterol levels can reflect the level of inflammation in vivo. A low level of LDL-C probably contributes to the pathogenesis of AF via enhancing inflammatory response, since chronic inflammation has been recognized as significantly related to the incidence and perpetuation of AF [59]. Third, lipids are the structural components of cell membranes. Balse et al. showed that changes in cholesterol levels may alter membrane structure and affect the function of ion channels and receptors. This can affect electrical gradient and resting potential across the membranes and potentially participate in the occurrence of AF [60].

Taken together, the results suggested that more attention should be paid to abnormal lipid levels by clinicians, not only as a risk factor for adverse cardiovascular outcomes, but also as a possible risk marker for AF, especially in elderly patients with higher DBP, TC or TG. These findings may provide additional evidence to distinguish high-risk AF patients to intervene before the symptoms appear in clinical practice and contribute to establishing personalized early interference strategies in primary healthcare facilities for people with a potential risk of AF.

### Comparisons with other studies and what does the current work add to the existing knowledge

In terms of novelty, a considerable number of studies have reported on the related genes of AF incidence, and it is the first time to explore the relationship between *APOE* gene polymorphism and AF among the Hakka population, provides new data for Hakka population research. A supplement to existing knowledge, the current study revealed that high levels of DBP, TC and TG were also significant independent risk factors for AF incidence in this study patients, not only closely linked with cardiovascular events.

## Strengths and limitations

This work is the first to investigate the association between the *APOE* gene polymorphism and lipid profile on the one side, and the risk of AF in the Hakka ethnic of southern China on the other side. The SNPs of the *APOE* gene (rs7412 and rs429358) were not significant risk for AF in the current study. By including and adjusting for the factors of comprehensive clinical characteristics, such as the echocardiographic parameters, laboratory test data and lipid profile, the results indicated that aged over 65 years, high levels of DBP, TC and TG were significant independent risk factors for AF in this study participants.

There are several limitations to this study. Firstly, for diseases with complex influencing factors, it has always been challenging to explore the relationship between a particular gene allele and such diseases. Hence, further genetic studies on other SNPs of the *APOE* gene are necessary to elucidate the precise association between the *APOE* gene polymorphism and AF. Plus, in addition to the moderating effect on the lipid profile levels, the antioxidant properties of the *APOE* genotypes should also be considered. Secondly, given the genetic diversity of different ethnic and regional populations and as a single-center study, these findings may not be applicable to other areas or ethnicities. Thirdly, lacking data on thyroid function and other echocardiographic parameters (e.g., left ventricular mass) might confound the results. Finally, owing to the retrospective study design, it is subject to the limitations inherent which were also noted in the previous study, possible selection bias has existed.

## Conclusions

In summary, the study suggested that well-established risk factors for CAD, older age as well as high levels of DBP, TC and TG were also significant independent risks for AF in the Hakka ethnic of southern Chinese. Furthermore, the levels of TG, TC, LDL-C and Apo-B depended significantly on AF and *APOE* allele groups, and statistically significant interactions between AF and *APOE* allele were observed in the above 4 variables, although the *APOE* gene SNPs (rs429358 and rs7412) were not significant independent risk factors for AF incidence in the study. The observed correlation needs to be further validated in future studies.

## Data Availability

Data and material would be supplied based on reasonable request.

## Abbreviations

AF: atrial fibrillation
GWAS: genome-wide association study
SBP: systolic blood pressure
DBP: diastolic blood pressure
CAD: coronary artery disease
HF: heart failure
LAD: left atrium diameter
LVDd: left ventricular end-diastolic diameter
LVSd: left ventricular end-systolic diameter
LVEF: left ventricular ejection fractions
TC: total cholesterol
TG: triglyceride
HDL-C: high-density lipoprotein cholesterol
LDL-C: low-density lipoprotein cholesterol
Apo-A1: apolipoprotein A1
Apo-B: apolipoprotein B
ALT: alanine aminotransferase
AST: aspartate aminotransferase
UN: urea nitrogen
Scr: serum creatinine
UA: uric acid
CRP: c-reactive protein
WBC: white blood cell
ACEI: angiotensin-converting enzyme inhibitor
ARB: angiotensin receptor blocker
BB: β-receptor blocker
MRA: mineralocorticoid antagonist
OR: odds ratio
CI: confidence interval
SD: standard deviation
SNP: single nucleotide polymorphism
CHD: coronary heart disease
PCR: polymerase chain reaction
LD: linkage disequilibrium
